# Long-read sequencing reveals the structural complexity of genomic integration of HBV DNA in hepatocellular carcinoma

**DOI:** 10.1038/s41525-021-00245-1

**Published:** 2021-10-12

**Authors:** Zhongling Zhuo, Weiqi Rong, Hexin Li, Ying Li, Xuanmei Luo, Ye Liu, Xiaokun Tang, Lili Zhang, Fei Su, Hongyuan Cui, Fei Xiao

**Affiliations:** 1 Peking University Fifth School of Clinical Medicine, Beijing, China; 2 The Key Laboratory of Geriatrics, Beijing Hospital, National Center of Gerontology, National Health Commission, Institute of Geriatric Medicine, Chinese Academy of Medical Sciences, Beijing, China; 3 Clinical Biobank, Beijing Hospital, National Center of Gerontology, National Health Commission, Institute of Geriatric Medicine, Chinese Academy of Medical Sciences, Beijing, China; 4 Department of Hepatobiliary Surgery, National Cancer Center/National Clinical Research Center for Cancer/Cancer Hospital, Chinese Academy of Medical Sciences (CAMS) and Peking Union Medical College (PUMC), Beijing, China; 5 Department of Surgery, Beijing Hospital, National Center of Gerontology, National Health Commission, Institute of Geriatric Medicine, Chinese Academy of Medical Sciences, Beijing, China

**Keywords:** Cancer genomics, Tumour virus infections

## Abstract

The integration of HBV DNA into the human genome can disrupt its structure in hepatocellular carcinoma (HCC), but the complexity of HBV genomic integration remains elusive. Here we applied long-read sequencing to precisely elucidate the HBV integration pattern in the human hepatocellular genome. The DNA library was sequenced using the long-read sequencing on GridION and PacBio Sequel II, respectively. The DNA and mRNA were sequenced using next-generation sequencing on Illumina NextSeq. BLAST (Basic Local Alignment Search Tool) and local scripts were used to analyze HBV integration patterns. We established an analytical strategy based on the long-read sequences, and analyzed the complexity of HBV DNA integration into the hepatocellular genome. A total of 88 integrated breakpoints were identified. HBV DNA integration into human genomic DNA was mainly fragmented with different orientations, rarely with a complete genome. The same HBV integration breakpoints were identified among the three platforms. Most breakpoints were observed at P, X, and S genes in the HBV genome, and observed at introns, intergenic sequences, and exons in the human genome. Tumor tissue harbored a much higher integrated number than the adjacent tissue, and the distribution of HBV integrated into human chromosomes was more concentrated. HBV integration shows different patterns between cancer cells and adjacent normal cells. We for the first time obtained the entire HBV integration pattern through long-read sequencing and demonstrated the value of long-read sequencing in detecting the genomic integration structures of viruses in host cells.

## Introduction

Hepatocellular carcinoma (HCC) ranks fifth in global cancer incidence and represents the third leading cause of cancer deaths^1^. Chronic hepatitis B virus (HBV) infection is a significant risk factor for the development of HCC in China, Southeast Asia, and sub-Saharan Africa^2^. Individuals with chronic HBV infection have an increased risk of cirrhosis or liver cancer^3^. Hepatitis B surface antigen (HBsAg) carriers have a 25−37 times higher risk of developing HCC than HsbAg negative controls^4,5^.

HBV infection-causing HCC may involve several main molecular mechanisms. The integration breakpoints change the HBV genome. The expression of HBV X protein (HBx) is involved in many intracellular signaling pathways related to cell proliferation and apoptosis, and both the C-terminal truncated HBx and the C-terminal region of HBx play essential roles in HCC^6^. Besides, the integration of HBV DNA into the host genome will change the expression and function of several essential genes that are related to the proliferation, differentiation, and survival of cells and the induction of chromosomal instability^7^. The most common HBV integration event is located in the *TERT*, which is thought to have an early cloning advantage during chronic HBV infection^8^. Recently, other common genes such as *KMT2B* and *CCNE1* have been identified as new genes previously unknown to have a causal role in cancer^9^. Besides, the accumulation of genetic damage caused by chronic inflammation mediated by regulatory T cells promotes the development of HCC.

Traditional techniques such as polymerase chain reaction (PCR), Southern blot, and Northern blot focus on known genes and typically investigate the integration of HBV in HCC in small samples^10–12^. With the availability of Next-Generation Sequencing (NGS), many studies have achieved a genome-wide investigation of HBV integration in HCC^7,13–16^. However, short-read sequencing is limited by its inability in sequencing full-length viral genomes directly, and the quality of its findings relies on many factors including the probe capture efficiency, sequencing depth, and analysis method. In addition, the requirement for rapid and accurate results for clinical application is not met with this method. Sine many studies^17,18^ have demonstrated the performance of long-read sequencing in detecting complex structural variations, this technique may be valuable for detecting HBV integration.

Based on the Nanopore and PacBio platforms, we utilized long-read sequences to detect the HBV complex integrated genome structure in HCC and its adjacent tissues. Using this new method, we obtained the entire HBV complex integrated structures for the first time. Furthermore, we also found differences in HBV integration patterns and gene expressions between HCC and its adjacent tissues.

## Results

### HBV integration breakpoints detected by Illumina platform

**Fig. 1:**
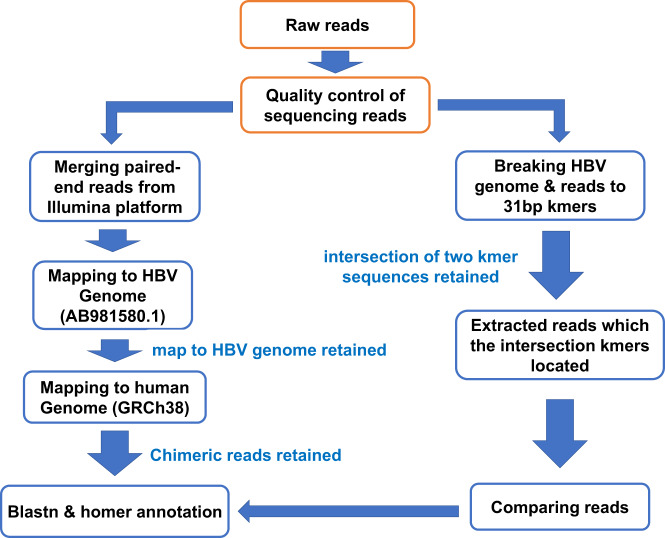
Workflow of HBV integration analysis. The raw data were first mapped to the HBV genotype C genome using BLAST. Reads mapped to the HBV genome were retained using local scripts and then mapped to the human genome. To verify the reliability of this workflow in detecting chimeric reads of HBV integration, another pipeline was used to compare the consistency of these two methods.

**Fig. 2:**
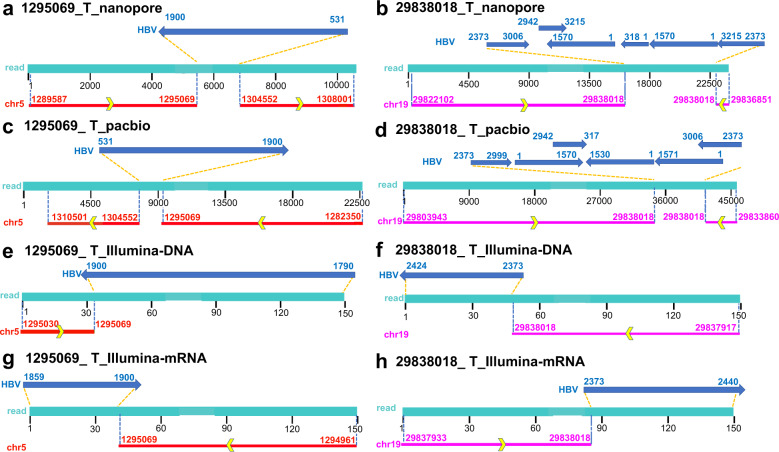
Locations of representative HBV-human chimeric reads in the HBV and human genome. **a**, **c**, **e**, **g** HBV: 1900, chr5: 1295069 detected in Nanopore, PacBio, Illumina (DNA), Illumina(mRNA) platforms. **b**, **d**, **f**, **h** HBV: 2373, chr19: 2983018 detected in Nanopore, PacBio, Illumina (DNA), Illumina(mRNA) platforms.

The DNA and mRNA of HCC and its adjacent tissues sequenced by Illumina platforms showed HBV breakpoints. The Illumina library was sequenced with NextSeq2000, and 90 Gb of original bases were generated, with a length of 150 bp. According to our workflow (Fig. 1), the sequences on the three platforms were analyzed, and the chimeric reads were analyzed using BLAST to determine the complex integrated genome structure. Illumina sequencing of the tumor yielded a total of 475 HBV-human chimeric reads, of which HBV was integrated into repeat region in 265 reads, and other 210 reads (only HBV breakpoints with a supporting read number ≥ 2) were kept for further analysis. Among them, HBV was integrated into intergenic nearest *CCNE1* in 153 reads (Fig. 2f), into *TERT* promoter-TSS in 17 reads (Fig. 2e), into *SYT14* intron 3 in 27 reads, and into *SERTAD4* intron 6 in seven reads. Also, HBV was integrated into *LINC01493* in two reads, into *MIR12125* in two reads, and into *MIR4457* in two reads. Illumina sequencing of the adjacent tissue demonstrated a total of 36 HBV-human chimeric reads, of which HBV was integrated into repeat region in 21 reads and the other ten reads (only HBV breakpoints with a supporting read number ≥ 2) were kept for further analysis. HBV was integrated into intergenic nearest *CCNE1* in two reads, into intergenic nearest *TENT5B* in two reads, into intergenic nearest *ARAP2* in two reads, and into 3′ UTR of *FGB* in four reads.

The mRNA library was sequenced with NextSeq2000. The tumor sequencing runs produced 6.7 Gb of bases, and sequencing of the adjacent tissue produced 12.1 Gb of bases, with a length of 150 bp. mRNA sequences of the tumor showed a total of 2003 HBV-human chimeric reads, of which HBV was integrated into repeat region in 1403 reads, and 600 reads (HBV breakpoints with a supporting read number ≥ 2) were kept for further analysis. Among them (HBV breakpoints with a supporting read number ≥ 50), HBV was integrated into intergenic nearest *CCNE1* in 99 reads (Fig. 2h), into *TERT* promoter-TSS in 66 reads (Fig. 2g), into SYT14 intron 3 in 110 reads, and into *MIR12125* in 192 reads. mRNA sequences of the adjacent tissue showed a total of 320 HBV-human chimeric reads, of which HBV was integrated into repeat region in 85 reads, and other 235 reads (only HBV breakpoints with a supporting read number ≥ 2) were kept for further analysis. Among them (HBV breakpoints with a supporting read number ≥ 10), HBV was integrated into *SERBP1* intron 13 in 17 reads, into intergenic nearest *SS18* in 16 reads, into *ZNF622* intron 3 in 11 reads, into intergenic nearest *LINC00364* in 10 reads, into *LOC105374060* intron 1 in 10 reads, into intergenic nearest *LINC02484* in 15 reads, and into intergenic nearest *LINC02065* in 20 reads.

### HBV integration breakpoints detected by Nanopore and PacBio platforms

The DNA of HCC and its adjacent tissues sequenced by Nanopore and PacBio platforms showed HBV complex integration patterns. The library was constructed and sequenced by ONT GridION. After low-quality reads (Q score < 7) were filtered out, the tumor sequencing run produced 5.39 Gb of bases and 927,710 reads from 1695 active wells, with the read length N50 being 15.3 kb. Sequencing of the adjacent tissue produced 8.41 Gb of bases and 2.19 × 10^6^ reads from 1630 active wells, with the read length N50 being 7.53 kb. A summary of the sequences in these three platforms is listed in Table 1. Nanopore sequencing of tumor detected ten breakpoints, of which HBV was integrated into intergenic nearest *CCNE1* in nine reads (Fig. 2b) and into *TERT* promoter-TSS in one read (Fig. 2a). Nanopore sequencing of adjacent tissue detected one breakpoint, which was HBV integrated into intergenic nearest *LINC02106*. We found that HBV was not integrated with a complete genome, but the genome was fragmented and integrated into the human genome in different orientations. In the nine HBV-*CCNE1* chimeric reads, the integrated HBV sequences had similar breakpoints, integration sequences, and integration orientations.

**Table 1:** The summary of the sequencing results.

Sample	Tumor	Adjacent tissue
HBV DNA viral load	>2.0 × 10^6^ IU/mL	>2.0 × 10^6^ IU/mL
***Nanopore sequencing results***		
Flowcell chemistry	R9.4.1	R9.4.1
Total bases	5.21 Gb	8.41 Gb
Total reads	927.71	2.19 × 10^6^
N50	15.3 Kb	7.63 Kb
HBV reads	10	2
HBV-human chimeric reads (repeat region)	0	0
HBV-human chimeric reads (non-repeat region)	10	1
***Pacbio sequencing results***		
Library	CLR	CLR
Total bases	110.36 Gb	75.02 Gb
Total reads	6.38 × 10^9^	6.52 × 10^9^
N50	24.27 Kb	13.10 Kb
HBV reads	131	9
HBV-human chimeric reads (repeat region)	21	1
HBV-human chimeric reads (non-repeat region)	92	8
***Illumina sequencing results***		
Total bases	90 Gb	90 Gb
reads length	150 bp	150 bp
HBV reads	7914	458
HBV-human chimeric reads (repeat region)	265	21
HBV-human chimeric reads (non-repeat region)	210	10
***RNA sequencing results***		
Total bases	6.7 G	12.1 G
reads length	150 bp	150 bp
HBV reads	29,091	13,052
HBV-human chimeric reads (repeat region)	1403	85
HBV-human chimeric reads (non-repeat region)	600	235

PacBio sequencing library was constructed and sequenced by PacBio Sequel II. The tumor sequencing runs produced 110.36 Gb of bases and 6.38 × 10^9^ reads after the low-quality reads (Q score < 7) were filtered out, with the read length N50 being 24.27 kb. Sequencing runs of adjacent tissue produced 75.02 Gb of bases and 6.52 × 10^9^ reads after the low-quality reads were filtered out. The read length N50 was 13.10 kb (Table 1). PacBio sequencing of tumor detected 128 breakpoints, of which HBV was integrated into repeat region in 21 reads, into intergenic nearest *CCNE1* (Fig. 2d) in 63 reads, into *TERT* promoter-TSS in one read (Fig. 2c), into *SYT14* intron 3 in 21 reads, into *SERTAD4* intron 6 in 18 reads, into *MIR12125* in three reads, and into MIR4457 in one read. PacBio sequencing of adjacent tissue detected breakpoints, of which HBV was integrated into intergenic nearest *CCNE1* in two reads and into *LOC149684* in six reads. We found that the HBV integration patterns detected by the PacBio platform were consistent with the Nanopore platform. HBV genome was fragmented and inserted into the human genome in different directions.

### HBV integration breakpoints in NGS and long-read sequencing

**Fig. 3:**
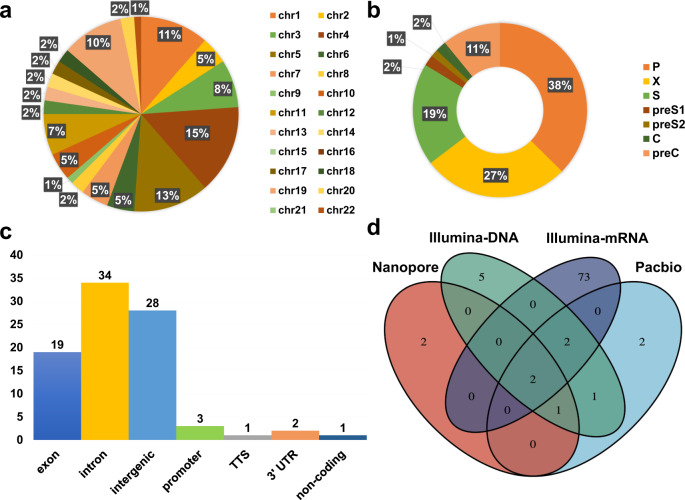
Distribution of integration breakpoints in human and HBV genome. **a** HBV integration breakpoints distribution in human chromosomes, different colors represent different chromosomes. **b** HBV integration breakpoints distribution in the HBV genome, different colors represent different genes of HBV. **c** HBV integration breakpoints functional locations in the human genome, different colors represent different regions of HBV. **d** overlap HBV integration breakpoints of four sequences, different colors represent different platforms of data.

The HBV breakpoints of HBV and human genome regions were analyzed based on three platforms, and the same sites were found between NGS and long-read sequencing data. HBV integration breakpoints and complex integration patterns were analyzed according to the workflow (Fig. 1). Finally, 88 integrated breakpoints (besides those integrated into the repeat region) were finalized (Supplemental Table 1). The distribution of all breakpoints on different chromosomes is shown in Fig. 3a. The HBV integration breakpoints (including HBV: 2373, chr19: 29838018, and HBV: 1900, chr5: 1295069) of chromosomes 19 and 5 were identified among three platforms. The distribution of integration breakpoints in the HBV genome is indicated in Fig. 3b. Consistent with a previous report^13^, most breakpoints were observed at P, X, and S genes in the tumor and adjacent tissue. As shown in Fig. 3c, most breakpoints were observed at introns, intergenic sequences, and exons in the human genome. The numbers of HBV-human chimeric read(s) identified in tumor and in adjacent tissue on different platforms were as follows: 10 and one read on Nanopore, 128 and 9 reads on PacBio, 475 and 31 reads on Illumina; in addition, mRNA sequencing identified 2003 and 320 reads. Although the throughput and sequencing depth of these three platforms were different, which resulted in different numbers of chimeric reads detected, we found that HBV: 2373, chr19: 29838018, and HBV: 1900, chr5: 1295069 were found in the tumor on all three platforms (Fig. 3d).

### Long-read sequencing discovers complex HBV integrated genome structures

While the short-read of Illumina was unable to sequence full-length HBV genomes directly, the long-read sequencing of Nanopore and PacBio gave the advantages of detecting complex integrated genome structures including the upstream and downstream of the HBV integrated into the human genome and the detailed integration sequences and orientations. In addition, microhomologies (MHs) were observed between the human genome and the HBV genome near integration breakpoints. Figure 2b reveals the complex HBV integration patterns in Nanopore sequencing. At chr19: 29838018, HBV first integrated 2373-3006 genomes in the forward orientation and then integrated forwards/backwards in 2942-3215, 1570-1, 318-1, 1570-1, and 3215-2373 genomes. Figure 2d shows that in PacBio sequencing, at chr19: 2983018, HBV first integrated 2373-2999 genomes forwards, and then integrated forwards/reverses with 1-1570, 2942-317, 1530-1, 1571-1, and 3006-2373 genomes. Figure 2a, c reveal that in Nanopore and PacBio sequencing, HBV integrated into chr5:1295069 and chr5:1304552. Figure 2e, g show that WGS and RNA sequences only detected chr5:1295069 breakpoint.

Among ten chimeric reads detected by Nanopore sequencing in the tumor, the single integrated fragment was found in three reads, and complex integrated fragments were found in seven reads (Supplemental Table 2). Among 92 chimeric reads detected by PacBio sequencing in the tumor, the single integrated fragment was found in 6 reads, and complex integrated fragments were found in 86 reads (Supplemental Table 3). Complex integrated fragments were found in all chimeric reads detected by Nanopore sequencing (Supplemental Table 2) and PacBio sequencing (Supplemental Table 3) in the adjacent tissue. Take the HBV: 2373, chr19: 29838018 for example: among nine chimeric reads detected by Nanopore sequencing in the tumor, all reads obtained the complete HBV integration structures. Among 63 chimeric reads detected by PacBio sequencing in the tumor, only two reads did not obtain the entire HBV integration structures, which demonstrated that long-read sequencing could discover complex HBV integrated genome structures.

**Fig. 4:**
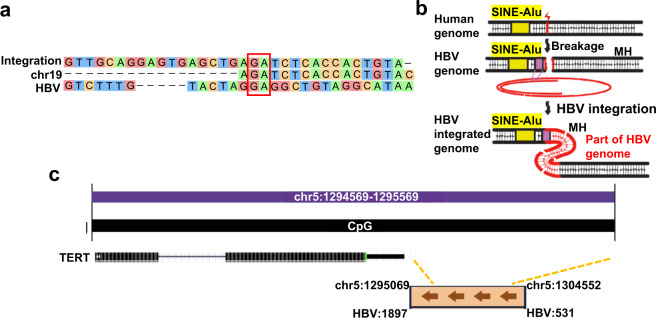
HBV integration mechanisms in HBV: 2373, chr19: 29838018, and HBV: 1900, chr5: 1295069. **a** Alignment of the sequence around the integration site between the human genome and the HBV genome. Different colors represent different nucleotide types. The red box represents the aligned nucleotides in the MH of the two reference sequences in the HBV integration site. **b** Schematic of a mechanism connecting breakage of the human genome around SINE-Alu motifs with the integration of the HBV genome. Yellow, SINE-Alu; purple, MH; red, HBV sequence. **c** HBV integration pattern in chr5. Yellow dotted line indicates Human chr5 breaks at nt 1295069 and nt 1304553 for HBV nt 1897 and nt 531 integration. the position of HBV integration breakpoints, which are near exon 1 of *TERT* and located in the CpG island.

We investigated HBV:2373, chr19:29838018, and HBV:1900, chr5:1295069 integration mechanisms. MHs were observed between the human genome and the HBV genome near integration breakpoints in HBV:2373, chr19:29838018 (Fig. 4a), which was similar to the HPV integration pattern reported^19^. Facilitated by the MHs flanking the breakpoint, HBV might hijack MH­mediated DNA repair pathways to fuse itself to the broken host genome and complete the integration process, as shown in Fig. 4b. HBV integrated into the CpG island near *TERT* exon1 in HBV: 1900, chr5: 1295069, leading to an evident upregulation of *TERT* (Fig. 4c).

### Comparison of HBV integration patterns and gene expression in tumor and adjacent tissue

**Fig. 5:**
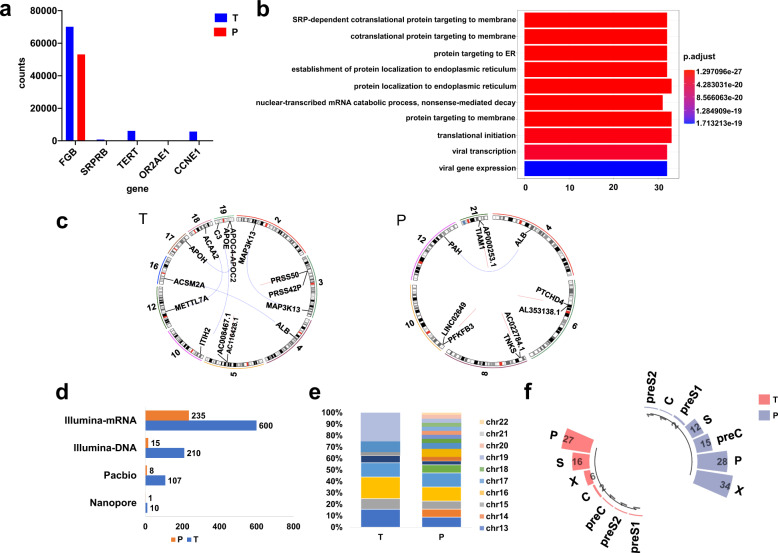
HBV integration patterns and gene expression in HCC tumor and adjacent tissue. **a** Gene expression in HBV integration affected genes (human breakpoint at promoter-TSS, 3′UTR, and TTS) in HCC tumor (blue) and adjacent tissue (red). **b** Differential expression gene enrichment pathways in tumor and adjacent tissue. Each bar depicts one enrichment pathway. **c** Fusion genes in tumor and adjacent tissue. Each line represents a breakpoint of fusion sites on different genes. **d** Chimeric reads numbers detected in tumor (blue) and adjacent tissue (red) in the three platforms. **e** HBV integration breakpoints distribution in human chromosomes in tumor and adjacent tissue, different colors represent different chromosomes. **f** HBV integration breakpoints distribution in the HBV genome in tumor (red) and adjacent tissue (blue).

Tumor and adjacent tissues showed different HBV integration patterns. All HBV integration breakpoints identified by three platforms are summarized into the final integrated site list (Supplemental Table 2). Following the known association between HBV infection and HCC development, tumor tissue harbored a much higher integrated number than the adjacent tissue (Fig. 5d). In addition to the repeat regions, a total of 88 integration breakpoints were found. HBV integration led to an evident upregulation of *TERT, CCNE1*, and *FGB* in the tumor (compared to the adjacent tissue) (Fig. 5a). The viral transcription and viral gene expression were up-regulated (Fig. 5b). More fusion genes were detected in the tumor than in adjacent tissue (Fig. 5c). The distribution of HBV integrated into human chromosomes in the tumor was more concentrated (Fig. 5e). The integration breakpoints of the HBV genome were mostly located in the P, X, and S genes (Fig. 5f).

## Discussion

Although the Illumina sequencing platform is highly sensitive in detecting viral infection status, it needs to be further improved in terms of detection accuracy and complex integrated genome structure identification. Nanopore and PacBio platforms have been proven to be fast yet accurate techniques for detecting pathogens in clinical samples with significantly longer sequencing lengths. However, the identification of virus integration breakpoints, especially HBV integration breakpoints, was seldom carried out. Most studies have shown that the error rate of the long-read sequencing is about 10–15%^20,21^, which greatly limits the application of these long-read sequencing platforms, including Nanopore and PacBio, in genome research. Here we combined two distinct HBV integration analysis workflows, extraction of chimeric reads after KMC (https://anaconda.org/bioconda/kmc) interruption, and extraction of chimeric reads after mapped to HBV and the human genome. We found that the chimeric reads of these two methods were consistent. Comparing the results from the Illumina sequences, we found that the HBV breakpoints in these three platforms could be verified, indicating that the long-read sequencing may effectively detect HBV integration. We for the first time obtained the entire HBV integration pattern through long-read sequencing, not based on algorithm predicting. We confirmed that HBV was not integrated with a complete genome; rather, the genome was fragmented and integrated into the human genome in different orientations continuously at the same site. Based on short-read sequencing analysis of inserted HBV fragment length prediction, the maximum HBV integration length (3215 bp) may be incorrect in the previous studies^22^. In another study^23^, TSD (a computational tool to study the complex structural variants) was applied to investigate HBV integration events in HBV infected PLC/PRF/5 cells based on the capture of HBV-specific probes using PacBio sequencing; it was found that the HBV integrated region was complex, consisting of several fragments and many HBV rearrangements. Peng^24^ found some forward and reverse complicated junctions of human-HBV chimeric reads in HCC patients, suggesting enriched MH exists between human and HBV genome sequences. MMEJ may be another important mechanism mediating virus integration processes^25^. Nevertheless, none of the sequences in these two studies and other NGS research covered the whole HBV integration region, which is critical for clarifying the HBV integration pattern including structural rearrangement, clonal expansion, and in-depth mechanisms. Using Nanopore and PacBio platforms, we obtained complex HBV integrated fragments in 111 sequences. We found MHs promoted the HBV integration, and HBV integration into the CpG island near *TERT* exon1 led to an evident upregulation of *TERT*. A more detailed HBV integration mechanism may be discovered using long-read sequencing.

It was previously reported that 40% of breakpoints observed were restricted to the HBV genome where the viral enhancer, X gene, and core gene are located^13^, and the sequences located in these regions participated in HBV integration into the host genome^6,13,26^. Our research demonstrated that 84% of the breakpoints were located in the region of HBV encoding polymerase protein, X protein, and env protein. In addition, 44% of HBV breakpoints were located at 1500–2000 base pairs on the HBV genome, close to either the direct repeat 1 (DR1) or direct repeat 2 (DR2), which verified the finding of a previous report^7^.

Ding^14^ and Toh^15^ concluded that HBV integration preferentially occurred in human chromosomes 17 and 10. Sung^13^ demonstrated that HBV integration was significantly enriched in other chromosomes including 4, 5, 8, 12, and Y. In our current study we found that HBV integration occurred on all autosomal chromosomes except chr15, chr16, chr21, priority on chr1, chr4, chr5, and chr19, which showed that our technology could reliably identify the chromosomes of HBV integration. Our data indicated that most integration breakpoints existed in the exons, intergenic sequences, and introns of the human genome, consistent with the previous study^13^. We also found that integration occurred in non-coding RNA (ncRNA), which plays many important functions (e.g., long ncRNA interaction with p53 protein)^27^. We found 46 genes were affected by HBV integration. HBV integration occurred in the promoter region of the *TERT* in the tumor, and such integration was not found in the adjacent tissue. In addition, the breakpoint of the promoter-TSS in TERT was detected in DNA sequencing, which was supported by RNA sequencing data. It was verified that the HBV integration, with a possible ‘cis-act’ on *TERT* transcription^28^, might up-regulate *TERT* transcription.

Our analysis showed the frequency of HBV integration was higher in the tumor than in the adjacent tissue. The chromosomes distributed at integration sites were more concentrated than in the adjacent tissue, and the same integration breakpoints were supported by more reads, which was consistent with the non-random integration model^28^. The positive selection might be carried out during the pathogenesis of liver cancer^28^. In the adjacent tissue, the integration model was more random. Jiang^7^ found that HBV integration frequently occurred in both tumor and nontumor hepatocytes, with distinct integration patterns. Clonal expansion of major integration sites, which carry hepatocytes, had been found specifically in the tumor samples but not in the matched liver samples^7^. However, a heterogeneous background population of cells harboring low-frequency viral integrations was detected both in the tumor and matched liver samples^7^. Similarly, a random integration model followed by a positive selection of major integration sites carrying hepatocytes during hepatocarcinogenesis resulted in more virus-integrated hepatocytes (clonally expanded subpopulation) in the tumor samples when compared to their matched nontumor counterparts^7^. In our current study, HBV was also prone to integrate into repetitive regions in the tumor than in adjacent tissues (Table 1), suggesting the preferential HBV integration into chromosomal repetitive regions may provide a selective advantage during tumorigenesis.

Since the sequencing was not based on HBV probe capture, the small number of HBV-human chimeric reads limited our analysis. Only one patient’s tumor and adjacent tissue were sequenced, which might result in sampling bias. Nevertheless, we obtained detailed HBV integration patterns. In our future studies, we will increase the depth of long-read sequencing and increase the sample size. On the other hand, due to the few chimeric reads obtained, the number of reads supported by the integration breakpoints was small, and many breakpoints were located in the repeat region of the human genome. It is not easy to verify by Sanger sequencing or other methods, but we did find some integration breakpoints among these three platforms, which confirmed the reliability of our results.

To the best of our knowledge, only two articles on long-read sequencing for identifying HBV integration patterns have been published^23,29^. Meng et al.^23^ used the HBV-specific probes to capture and enriched the DNA pieces that carry HBV sequences, and the library was sequenced by PacBio Sequel. TSD was applied to investigate HBV integration events in HBV infected PLC/ PRF/5 cells and discovered 9 HBV integration events. The authors noticed that such an HBV-rearranged sequence was about 2400 bp and no single PacBio read covered the whole region, which confirmed our findings in another perspective. Peneau et al.^29^ discovered the complex integration sites of three patients’ tumors reconstructed by long-read length (CLR and CCS) and optical mapping. However, in two integration events they reconstructed, HBV integrated only one fragment in the human genome. Different from their research, we found that the integration of HBV DNA into human genomic DNA is mainly fragmented in different orientations (Fig. 2b, d).

In summary, compared with Illumina sequencing and long-read sequencing based on HBV probe capture, long-read sequencing without HBV probe capture has apparent advantages in detecting complex HBV integrated genome structures. The long-read sequencing we developed for HBV integration patterns will become an essential tool in future research or related clinical applications. It will be particularly helpful in elucidating HBV integration patterns including structural rearrangement, clonal expansion, and its in-depth mechanisms.

## Methods

### Sample collection

In 2020, the tumor and a pair of adjacent tissue from a patient with Ib HCC were resected at the Cancer Hospital of the Chinese Academy of Medical Sciences. Specimens were immediately cryopreserved at −80 °C following resection. The patient’s blood test results were HBsAg-positive, HBeAb-positive, and HBcAb-positive, and the HBV genotype C DNA viral loads of both tumor tissue and adjacent tissue were higher than 2.0 × 10^6^ IU/mL.

### Ethical approval

This study complies with the “Ministry of Science and Technology (MOST) Guidelines on the Review and Approval of Human Genetic Resources” and has officially approved the export of human genetic materials or data from China. This study was approved by the institutional review board of Beijing hospital. An informed consent form was collected from this patient. All authors had access to the study data and had reviewed and approved the final manuscript.

### Nanopore sequencing

The tissues were extracted using the MagAttract HMW DNA kit (Qiagen). Nanodrop 2000 and Qubit dsDNA HS analysis kits (both from Thermo Fisher Scientific) were used to quantify double-stranded (ds) DNA. The DNA was purified using AMPure XP (Beckman Coulter), and the DNA concentration was measured using Qubit^®^3.0 fluorometer (Life Technologies). The library was sequenced using the Nanopore sequencing platform GridION, R9.4.1 chip (ONT). After the quality inspection was completed on the chip according to the instructions, a library was constructed according to the operating procedure of the library building kit, and samples were added to the chip. The ONT sequencer control software MinKNOW (v3.5.10) was used to collect the raw electronic signal data. The Guppy (v3.2.6) software was used to convert the FAST5 files to FASTQ files.

### PacBio sequencing

Genomic DNA of the tumor and a pair of adjacent tissue were extracted using QIAamp DNA Mini Kit (Qiagen). The integrity of the DNA was determined with the Agilent 4200 Bioanalyzer (Agilent Technologies). Eight micrograms of genomic DNA were sheared using g-Tubes (Covaris) and concentrated with AMPure PB magnetic beads. Each SMRT bell library was constructed using the SMRTbell Express Template Prep Kit 2.0 (Pacific Biosciences). The constructed libraries were size-selected on a BluePippinTM system (Sage Science) for 30 kb molecules, followed by the primer annealing and the binding of SMRT bell templates to polymerases with the Sequel II Binding and Internal Control Kit 1.0 Kit (Pacific Biosciences). Sequencing was carried out on the Pacific Bioscience Sequel II platform with Sequel II Sequencing 1.0 Bundle (Pacific Biosciences) at Annoroad Gene Technology company.

### Illumina sequencing

According to the instructions of the manufacturer, the QIAamp DNA Mini Kit (Qiagen) was used to extract genomic DNA and the Dynabeads^TM^ mRNA Purification Kit (Invitrogen) was used to extract mRNA from total RNA. The library was quantified with the Qubit dsDNA HS Assay Kit using a Qubit 3.0 fluorometer (both from Thermo Fisher Scientific) for quality analysis before sequencing. The library was prepared according to the Illumina sample preparation guide. DNA libraries were sequenced using the NextSeq platform (Illumina) with 150 bp paired-end reads.

### HBV integration analysis

FastQC (https://github.com/s-andrews/FastQC) software was performed for quality control of sequences. FLASH (https://ccb.jhu.edu/software/FLASH) was used to merge paired-end reads from next-generation sequencing experiments. Seqkit2 (https://bioinf.shenwei.me/seqkit) was performed to convert FASTQ files to FASTA files. We detected HBV integration breakpoints using BLAST^30^ and local scripts in all data from three platforms. The workflow was shown in Fig. 1. The raw data were first mapped to the HBV genotype C genome (AB981580.1) using BLAST. Reads mapped to the HBV genome were retained using local scripts and then mapped to the human genome (GRCh38). For each sequence, we further filtered the files by selecting all viral HSPs and the first three human HSPs. We visually inspected these files to identify sequences containing human-virus-human or human-virus connections. Chimeric reads (read sequences that were partially aligned to the human genome and partially to the HBV genome) were retained and the complex integrated genome structure was analyzed. HBV integration breakpoints were annotated using homer (https://anaconda.org/bioconda/homer). To increase the length of the HBV integration sequences identified in the Illumina platform, we combined the paired-end reads, analyzed the extended reads, and uncombined reads separately.

To verify the reliability of this workflow in detecting chimeric reads of HBV integration, we also used another pipeline to compare the consistency of these two methods. We used KMC software to break the HBV genome and sequence data into 31 bp kmers. The local scripts were used to obtain the intersection of the two group kmers. Furthermore, we extracted the reads where the intersection kmers were located. HBV breakpoints with a supporting read number ≥ 2 were regarded as highly confident HBV integration sites in the subsequent analysis in Illumina sequencing.

### Gene expression analysis

After mapping the reads to the human genome (GRCh37, hg19) using STAR (https://github.com/alexdobin/STAR), a normalized gene expression matrix file was obtained through HTSeq (https://pypi.org/project/HTSeq) and stringtie (ccb.jhu.edu/software/stringtie/). NOIseq (http://bioinfo.cipf.es/noiseq) was performed for differential expression genes analysis in no replicate sample. The threshold of differential expression genes was set as foldChange > 2 and adjusted *P*-value < 0.05. The “clusterProfiler”^29^ R package was used for Gene Ontology enrichment analysis^29^ on genes or nearest genes that the HBV breakpoints located. Enriched Gene Ontology terms with adjusted *P*-value < 0.05 were considered statistically significant. STAR-Fusion (http://star-fusion.github.io/) was performed to identify fusion transcripts from RNA sequences data.

### Reporting summary

Further information on research design is available in the Nature Research Reporting Summary linked to this article.

## Supplementary information

Supplementary Information

Supplemental Data 1

Supplemental Data 2

Supplemental Data 3

Reporting Summary

## Data Availability

The sequencing data has been submitted to the Genome Sequence Archive and is available under the accession number HRA001037.
