# Supplementary material for: Long-read sequencing reveals the structural complexity of genomic integration of HBV DNA in hepatocellular carcinoma
Source: NPJ Genom Med. 2021 Oct 12;6:84. doi: 10.1038/s41525-021-00245-1 (PMC8511263; doi:10.1038/s41525-021-00245-1)
Supplement: Supplementary file 1 — Supplementary Information [file 41525_2021_245_MOESM1_ESM.pdf]

Supplemental Data 1 The summary of identified HBV integration breakpoints.

Supplemental Data 2 The summary of HBV integration fragments in Nanopore sequences.

Supplemental Data 3 The summary of HBV integration fragments in PacBio sequences.
