# Supplementary material for: Long-read sequencing reveals the structural complexity of genomic integration of HBV DNA in hepatocellular carcinoma
Source: NPJ Genom Med. 2021 Oct 12;6:84. doi: 10.1038/s41525-021-00245-1 (PMC8511263; doi:10.1038/s41525-021-00245-1)
Supplement: Supplementary file 5 — Reporting Summary [file 41525_2021_245_MOESM5_ESM.pdf]

nature portfolio

Corresponding author(s): Hongyuan Cui & Fei Xiao

Last updated by author(s): Aug 3, 2021

Reporting Summary

Nature Portfolio wishes to improve the reproducibility of the work that we publish. This form provides structure for consistency and transparency in reporting. For further information on Nature Portfolio policies, see our Editorial Policies and the Editorial Policy Checklist.

Statistics

For all statistical analyses, confirm that the following items are present in the figure legend, table legend, main text, or Methods section.

n/a	Confirmed	
☐	☒	The exact sample size (*n*) for each experimental group/condition, given as a discrete number and unit of measurement
☐	☒	A statement on whether measurements were taken from distinct samples or whether the same sample was measured repeatedly
☐	☒	The statistical test(s) used AND whether they are one- or two-sided<br>*Only common tests should be described solely by name; describe more complex techniques in the Methods section.*
☐	☒	A description of all covariates tested
☐	☒	A description of any assumptions or corrections, such as tests of normality and adjustment for multiple comparisons
☐	☒	A full description of the statistical parameters including central tendency (e.g. means) or other basic estimates (e.g. regression coefficient) AND variation (e.g. standard deviation) or associated estimates of uncertainty (e.g. confidence intervals)
☐	☒	For null hypothesis testing, the test statistic (e.g. *F*, *t*, *r*) with confidence intervals, effect sizes, degrees of freedom and *P* value noted<br>*Give P values as exact values whenever suitable.*
☒	☐	For Bayesian analysis, information on the choice of priors and Markov chain Monte Carlo settings
☒	☐	For hierarchical and complex designs, identification of the appropriate level for tests and full reporting of outcomes
☒	☐	Estimates of effect sizes (e.g. Cohen's *d*, Pearson's *r*), indicating how they were calculated

Our web collection on statistics for biologists contains articles on many of the points above.

Software and code

Policy information about availability of computer code

Data collection	The DNA library was sequenced using the long-read sequencing on GridION and PacBio Sequel II, respectively. The DNA and mRNA were sequenced using next-generation sequencing on Illumina NextSeq
Data analysis	HBV integration analysis<br>FastQC (https://github.com/s-andrews/FastQC) software was performed for quality control of sequences. FLASH (https://ccb.jhu.edu/software/FLASH) was used to merge paired-end reads from next-generation sequencing experiments. Seqkit2 (https://bioinf.shenwei.me/seqkit) was performed to convert FASTQ files to FASTA files. We detected HBV integration breakpoints using BLAST30 and local scripts in all data from three platforms. The workflow was shown in Figure 1. The raw data were first mapped to the HBV genotype C genome (AB981580.1) using BLAST. Reads mapped to the HBV genome were retained using local scripts and then mapped to the human genome (GRCh38). For each sequence, we further filtered the files by selecting all viral HSPs and the first three human HSPs. We visually inspected these files to identify sequences containing human-virus-human or human-virus connections. Chimeric reads (read sequences that were partially aligned to the human genome and partially to the HBV genome) were retained and the complex integrated genome structure was analyzed. HBV integration breakpoints were annotated using homer (https://anaconda.org/bioconda/homer). To increase the length of the HBV integration sequences identified in the Illumina platform, we combined the paired-end reads, analyzed the extended reads, and uncombined reads separately.<br>To verify the reliability of this workflow in detecting chimeric reads of HBV integration, we also used another pipeline to compare the consistency of these two methods. We used KMC software to break the HBV genome and sequence data into 31bp kmers. The local scripts were used to obtain the intersection of the two group kmers. Furthermore, we extracted the reads where the intersection kmers were located. HBV breakpoints with a supporting read number ≥ 2 were regarded as highly confident HBV integration sites in the subsequent analysis in Illumina sequencing.<br>Gene expression analysis<br>After mapping the reads to the human genome (GRCh37, hg19) using STAR (https://github.com/alexdobin/STAR), a normalized gene expression matrix file was obtained through HTSeq (https://pypi.org/project/HTSeq) and stringtie (ccb.jhu.edu/software/stringtie/). NOIseq (http://bioinfo.cipf.es/noiseq) was performed for differential expression genes analysis in no replicate sample. The threshold of differential

expression genes was set as foldChange > 2 and adjusted P-value < 0.05. The "clusterProfiler"29 R package was used for Gene Ontology enrichment analysis29 on genes or nearest genes that the HBV breakpoints located. Enriched Gene Ontology terms with adjusted P-value < 0.05 were considered statistically significant. STAR-Fusion (http://star-fusion.github.io/) was performed to identify fusion transcripts from RNA sequences data.

For manuscripts utilizing custom algorithms or software that are central to the research but not yet described in published literature, software must be made available to editors and reviewers. We strongly encourage code deposition in a community repository (e.g. GitHub). See the Nature Portfolio guidelines for submitting code & software for further information.

Data

Policy information about availability of data

All manuscripts must include a data availability statement. This statement should provide the following information, where applicable:

- Accession codes, unique identifiers, or web links for publicly available datasets
- A description of any restrictions on data availability
- For clinical datasets or third party data, please ensure that the statement adheres to our policy

The sequencing data has been submitted to the Genome Sequence Archive and is available under the accession number HRA001037.

Field-specific reporting

Please select the one below that is the best fit for your research. If you are not sure, read the appropriate sections before making your selection.

☒ Life sciences ☐ Behavioural & social sciences ☐ Ecological, evolutionary & environmental sciences

For a reference copy of the document with all sections, see nature.com/documents/nr-reporting-summary-flat.pdf

Life sciences study design

All studies must disclose on these points even when the disclosure is negative.

Sample size	the tumor and a pair of adjacent tissue from a patient with Ib HCC
Data exclusions	Not Applicable
Replication	Not Applicable
Randomization	Not Applicable
Blinding	Not Applicable

Reporting for specific materials, systems and methods

We require information from authors about some types of materials, experimental systems and methods used in many studies. Here, indicate whether each material, system or method listed is relevant to your study. If you are not sure if a list item applies to your research, read the appropriate section before selecting a response.

Materials & experimental systems

n/a	Involved in the study	
☒	☐	Antibodies
☒	☐	Eukaryotic cell lines
☒	☐	Palaeontology and archaeology
☒	☐	Animals and other organisms
☐	☒	Human research participants
☒	☐	Clinical data
☒	☐	Dual use research of concern

Methods

n/a	Involved in the study	
☒	☐	ChIP-seq
☒	☐	Flow cytometry
☒	☐	MRI-based neuroimaging

Human research participants

Policy information about studies involving human research participants

Population characteristics	a patient with Ib HCC. The patient's blood test results were HBsAg-positive, HBeAb-positive, and HBcAb-positive, and the HBV genotype C DNA viral loads of both tumor tissue and adjacent tissue were higher than 2.0 × 106 IU/mL.

Recruitment	Not Applicable
Ethics oversight	This study was approved by the institutional review board of Beijing hospital. An informed consent form was collected from this patient. All authors had access to the study data and had reviewed and approved the final manuscript.

Note that full information on the approval of the study protocol must also be provided in the manuscript.
